# Blood Glucose Homeostasis in the Course of Partial Pancreatectomy – Evidence for Surgically Reversible Diabetes Induced by Cholestasis

**DOI:** 10.1371/journal.pone.0134140

**Published:** 2015-08-06

**Authors:** Florian Ehehalt, Dorothée Sturm, Manuela Rösler, Marius Distler, Jürgen Weitz, Stephan Kersting, Barbara Ludwig, Uta Schwanebeck, Hans-Detlev Saeger, Michele Solimena, Robert Grützmann

**Affiliations:** 1 Department of GI, Thoracic and Vascular Surgery, University Hospital Carl Gustav Carus, TU Dresden, Germany; 2 Paul Langerhans Institute Dresden of the Helmholtz Center Munich at University Hospital Carl Gustav Carus and Faculty of Medicine, TU Dresden, Germany; 3 German Center for Diabetes Research (DZD e.V.), Neuherberg, Germany; 4 Coordination Center for Clinical Trials, TU Dresden, Germany; 5 Department of Medicine III, University Hospital Carl Gustav Carus, TU Dresden, Germany; 6 Max Planck Institute of Molecular Cell Biology and Genetics, Dresden, Germany; University Hospital Oldenburg, GERMANY

## Abstract

**Background and Aim:**

Partial pancreatic resection is accompanied not only by a reduction in the islet cell mass but also by a variety of other factors that are likely to interfere with glucose metabolism. The aim of this work was to characterize the patient dynamics of blood glucose homeostasis during the course of partial pancreatic resection and to specify the associated clinico-pathological variables.

**Methods:**

In total, 84 individuals undergoing elective partial pancreatic resection were consecutively recruited into this observational trial. The individuals were assigned based on their fasting glucose or oral glucose tolerance testing results into one of the following groups: (I) deteriorated, (II) stable or (III) improved glucose homeostasis three months after surgery. Co-variables associated with blood glucose dynamics were identified.

**Results:**

Of the 84 participants, 25 (30%) displayed a normal oGTT, 17 (20%) showed impaired glucose tolerance, and 10 (12%) exhibited pathological glucose tolerance. Elevated fasting glucose was present in 32 (38%) individuals before partial pancreatic resection. Three months after partial pancreatic resection, 14 (17%) patients deteriorated, 16 (19%) improved, and 54 (64%) retained stable glucose homeostasis.

Stability and improvement was associated with tumor resection and postoperative normalization of recently diagnosed glucose dysregulation, preoperatively elevated tumor markers and markers for common bile duct obstruction, acute pancreatitis and liver cell damage. Improvement was linked to preoperatively elevated insulin resistance, which normalized after resection and was accompanied by a decrease in fasting- and glucose-stimulated insulin secretion.

**Conclusions:**

Surgically reversible blood glucose dysregulation diagnosed concomitantly with a (peri-) pancreatic tumor appears secondary to compromised liver function due to tumor compression of the common bile duct and the subsequent increase in insulin resistance. It can be categorized as “cholestasis-induced diabetes” and thereby distinguished from other forms of hyperglycemic disorders.

## Introduction

The endocrine function of the pancreas is essential for blood glucose regulation. Total pancreatectomy leads to the complete absence of insulin and glucagon production, which is followed by severe dysregulation of blood glucose levels. In contrast, partial pancreatic resection is accompanied by endocrine consequences that are more complex.

It remains unclear whether diabetes is a predisposing factor for pancreatic neoplasms or a consequence of pancreatic pathology, although a body of evidence points to a time-dependent association of new-onset diabetes and pancreatic ductal adenocarcinoma (PDAC) [1–4]. Under certain undefined circumstances, the resection of pancreatic carcinoma can be followed by a postoperative improvement in glucose homeostasis in diabetic patients, which is accompanied by an increase in peripheral insulin sensitivity [5]. These observations have led to the hypothesis that a diabetogenic factor might result from the tumor, specifically pancreatic ductal adenocarcinomas [6,7]. However, in the course of pancreatic resection, other variables, such as dynamics in body weight, alterations of the anatomy of the alimentary tract and changes of liver function and acute or chronic pancreatic inflammation, may interfere with blood glucose homeostasis. In this light, the hypothesis that diabetes is induced by a tumor-intrinsic factor appears reductionist.

While all types of partial pancreatic resections result in a loss of pancreatic endocrine tissue, these interventions may be performed with or without changes in the gastro-duodeno-jejunal passage (PPPD/Whipple procedure versus pancreatic left resections or duodenum-preserving head resections, respectively). Alterations of the gastro-duodeno-jejunal passage are implemented during bariatric gastro-intestinal bypass procedures to positively modify glucose regulation. Better glucose control after bariatric surgery seems mainly attributable to weight loss and the resulting improvement of insulin sensitivity. The role of increased incretin secretion (i.e., glucagon-like peptide 1), and consequently improvement of plasma insulin levels, have been documented for bypass procedures. However, no positive effects on postprandial peaks of plasma glucose have been observed. Additionally, the data concerning the effect of altered secretion of glucagon or glucose-dependent insulinotropic peptide after bariatric bypass procedures on glucose control are not conclusive [8]. For example, studies on the associations between altered anatomy of the upper gastrointestinal tract after partial pancreatic resection and incretin responses and blood glucose dynamics have yielded inconclusive results [1,9].

A common presentation of pancreatic pathologies is jaundice, which occurs due to the obstruction of the common bile duct and is the classical symptom of pancreatic head malignancies and consequent impaired liver function. In the context of chronic liver disease, markers for common bile duct obstruction and liver cell damage are associated with the development of insulin resistance, metabolic syndrome and type 2 diabetes [10–17]. Some molecular mechanisms for liver-mediated insulin resistance have already been characterized [18].

The influence of bile flow restoration by pancreatic surgery on glucose metabolism has not yet been investigated, and most published studies have not commented on the influence of the above-mentioned co-factors on blood glucose homeostasis after major upper gastrointestinal surgery. Furthermore, previous studies have often been limited by highly selective cohorts, metabolic work-ups based on medical history alone, and/or short follow-up periods.

To elucidate the complex consequences of different pancreatic pathologies and surgical interventions on blood glucose homeostasis, we designed an observational study that included all patients undergoing partial pancreatic resections. Glucose homeostasis was determined before and three months after surgery. The aim was to identify variables associated with clinically relevant dynamics in blood glucose homeostasis and to clarify the underlying mechanisms leading to these dynamics.

## Methods

### Study design and recruitment

The present investigation was conducted in the outpatient clinic for pancreatic surgery at the University Hospital Carl Gustav Carus of the TUD, which is a tertiary referral center for pancreatic disease. The local ethical committee (“*Ethik-Kommission der Medizinischen Fakultät Carl Gustav Carus der Technischen Universität Dresden*”) approved the study (approval number: EK151062008), and all of the participants provided their written informed consent.

During the period from 02/2010 to 03/2013, 84 consecutive participants who were scheduled for partial pancreatic resections were included in the study, independent of their indication for surgery. Patients younger than 18 years were excluded. Because a classic Whipple procedure is accompanied by loss of the pylorus, which may predispose patients to dumping syndrome upon glucose ingestion, patients in this category were excluded. A follow-up period of three months was chosen to allow for the resolution of metabolic disturbances following major abdominal surgery.

Immediately prior to and three months after surgery, the patients’ detailed medical history was documented and their body mass index (BMI) was determined. Furthermore, their HbA1c and fasting glucose levels were measured, and if physiological values (<7.0 mmol/L) were obtained, a 120-minute oral glucose tolerance test (oGTT) using 75 g (300 ml) of a liquid glucose solution was performed, according to international standards. Plasma values for glucose, as well as their serum values for insulin, proinsulin and C-peptide, were measured at 0, 60 and 120 minutes. Fasting potassium and magnesium values were also determined to exclude false-positive test results.

### Severity grades of blood glucose dysregulation and dynamics

To quantify the clinically relevant changes in blood glucose regulation during the course of pancreatic surgery, participants were assigned to one of the following categories related to the severity of blood glucose dysregulation prior to and three months after surgery:

1. normal oGTT (NGT; fasting glucose <7.0 mmol/l; 120 min oGTT value <7.8 mmol/l);
2. impaired oral glucose tolerance (IGT; fasting glucose <7.0 mmol/l; 120 min oGTT value 7.8–11.1 mmol/l);
3. diabetes based on pathological oral glucose tolerance (PGT; fasting glucose <7.0 mmol/l; 120 min oGTT value >11.1 mmol/l)
4. diabetes based on elevated fasting glucose (fasting glucose >7.0 mmol/l) or anti-diabetic medication.

Based on these categories, the patients were included into the three following groups:

1. improvement of blood glucose homeostasis, if the post-operative category was less than the pre-operative category;
2. stable blood glucose homeostasis, if the post-operative category equaled the pre-operative category;
3. deterioration of blood glucose homeostasis, if the post-operative category was greater than the pre-operative category.

### Assessment of co-variables

We determined the following parameters according to standard operational procedures prior to and three months after surgery:

Glucose, insulin, C-peptide, proinsulin, HbA1c, C-reactive protein (CRP), gamma-glutamyltransferase (γGT), alkaline phosphatase (ALP), bilirubin, P-amylase, lipase, hemoglobin, leukocytes, total protein, albumin, triglycerides, cholesterol, high-density lipoprotein cholesterol (HDLC), low-density lipoprotein cholesterol (LDLC), carcinoembryonic antigen (CEA), carbohydrate antigen 19–9 (CA19-9), alanine aminotransferase (ALAT), aspartate aminotransferase (ASAT), potassium and magnesium.

Insulin sensitivity was estimated in the fasting state, using the homeostatic model assessment of insulin resistance (HOMA-IR) index. β-cell function was estimated using the homeostatic model assessment of β-cell function (HOMA-B) index [19,20].

The indices were calculated using mathematical estimation in a single sample, as follows:

$$\text{HOMA}-\text{IR}:\;\left({\text{I}}_{0}\cdot \;{\text{G}}_{0}\right)/22.5$$

where I_0_ = fasting insulin in μU/mL and G_0_ = fasting plasma glucose in mmol/L;

$$\text{HOMA}-\text{B}:\;\left(20\cdot {\text{I}}_{0}\right)/\left({\text{G}}_{0}–3.5\right)$$

where I_0_ = fasting insulin in μU/ml and G_0_ = fasting plasma glucose in mmol/L.

Based on the results of the histopathological examinations, we categorized the patients into three groups as follows: subjects with malignant tumors, subjects with benign tumors / lesions and subjects with chronic pancreatitis (CP).

Based on the surgical procedures, we categorized the patients into three groups as follows: subjects undergoing PPPD, subjects undergoing duodenum-preserving pancreatic head resections (DPPHR) and subjects undergoing pancreatic left resections.

The width of the pancreatic duct was either determined preoperatively through imaging (ultrasonography/CT/MRCP) or was estimated during the operation by the surgeon. A diameter greater than 3 mm was classified as pathological.

For all patients who underwent an oral glucose tolerance test, the areas under the curve (AUCs) for glucose, insulin, C-peptide and proinsulin were calculated using the trapezoid rule for oGTT-values at 0, 60 and 120 minutes.

### Statistics

For the comparison of the three groups (i.e., deterioration, stability, and improvement) based on the categorical variables (one time point), a two-sided χ^2^-test was used. Exact p-values <.05 were defined as significant. For the comparison of continuous variables among the three groups at one time point, one-way ANOVA was used, with p<.05 considered to be significant. For the comparison of continuous variables within one group at two time points (i.e., pre- and postoperative), the two-sided T-test for paired samples was used, and exact p-values <.05 were considered to be significant.

All of the statistical analyses were performed using IBM SPSS for Macintosh, Version 19.

## Results

In total, 84 participants were enrolled. The mean age of the participants was 60.8 years (range, 30–79).

At the preoperative assessment, 25/84 (30%) of the patients had normal oGTT, while 17/84 (20%) showed IGT, 10/84 (12%) presented with diabetes diagnosed by a pathological glucose tolerance (PGT), and 32/84 (38%) were diabetic based on their fasting glucose levels and/or anti-diabetic medications.

The postoperative histological examination revealed 24 cases of chronic pancreatitis (CP) and 15 benign (peri-) pancreatic tumors, specifically, five serous cystic neoplasms, two ampullary adenomas, three intraductal papillary mucinous neoplasms, one non-functional neuro-endocrine tumor, one autoimmune pancreatitis, one case of endometriosis, one gastrointestinal stroma tumor and one case of duodenal heterotopy. The 45 malignant tumors included 24 pancreatic ductal adenocarcinomas, eight ampullary carcinomas, four carcinomas of the distal common bile duct, one malignant gastrointestinal stroma tumor, one metastasis of renal cell carcinoma, five non-functional neuro-endocrine carcinomas and one mucinous adenocarcinoma.

### Categorical variables

When surgery was performed due to CP, 83% of patients did not change their glucose regulation category, 17% deteriorated, and none improved. This distribution contrasted with the group of patients undergoing surgery for tumor removal (p = .02). Only 47% of patients with benign disease and 60% with malignant disease remained stable, while 33% of patients with benign lesions exhibited deteriorated glucose homeostasis and 29% of patients with malign tumors improved after surgery (Table 1).

**Table 1:** Distribution of the categorical variables based on the dynamics of blood glucose homeostasis after partial pancreatectomy.

Variable		Deterioration (n = 14)	Stability (n = 54)	Improvement (n = 16)	p-value (χ^2^-Test)
**age (n = 84)**	<60 (n = 40)	6 (15%)	32 (80%)	2 (5%)	**.004**
	≥60 (n = 44)	8 (18%)	22 (50%)	14 (32%)	
**gender (n = 84)**	male (n = 57)	9 (16%)	38 (67%)	10 (17%)	.80
	female (n = 27)	5 (19%)	16 (59%)	6 (22%)	
**Preoperative weight loss (n = 84)**	no (n = 53)	11 (21%)	34 (64%)	8 (15%)	.27
	yes (>5 kg) (n = 31)	3 (10%)	20 (64%)	8 (26%)	
**surgical procedure (n = 84)**	left resection (n = 13)	3 (23%)	10 (77%)	0 (0%)	.42
	PPPD (n = 60)	9 (15%)	37 (62%)	14 (23%)	
	DPPHR (n = 11)	2 (16%)	7 (64%)	2 (20%)	
**Pancreatic duct (n = 83)**	normal (<3 mm) (n = 38)	7 (18%)	24 (64%)	7 (18%)	.94
	dilated (n = 45)	7 (16%)	29 (64%)	9 (21%)	
**Histology(n = 84)**	cP (n = 24)	4 (17%)	20 (83%)	0 (0%)	**.02**
	malignant (n = 45)	5 (11%)	27 (60%)	13 (29%)	
	benign (n = 15)	5 (33%)	7 (47%)	3 (20%)	
**diagnosis of dysregulation (n = 56)**	<6 month preoperative (n = 31)	7 (23%)	10 (32%)	14 (45%)	**<.001**
	≥6 month preoperative (n = 25)	0 (0%)	24 (96%)	1 (4%)	
**preoperative category (n = 84)**	NGT (n = 25)	7 (28%)	18 (72%)	0 (0%)	**<.001**
	IGT (n = 17)	6 (35%)	4 (24%)	7 (41%)	
	PGT (n = 10)	1 (10%)	3 (30%)	6 (60%)	
	Elevated fasting glucose/ADM (n = 32)	0 (0%)	29 (91%)	3 (9%)	
**postoperative category (n = 84)**	NGT (n = 29)	0 (0%)	18 (62%)	11 (38%)	**<.001**
	IGT (n = 14)	5 (36%)	4 (28%)	5 (36%)	
	PGT (n = 10)	7 (70%)	3 (30%)	0 (0%)	
	Elevated fasting glucose/ADM (n = 31)	2 (7%)	29 (93%)	0 (0%)	

Percentages are expressed in parenthesis in each row, and the p-values were obtained using a χ^2^-test. ADM = anti-diabetic medication; CP = chronic pancreatitis; DPPHR = duodenum-preserving pancreatic head resection; IGT = impaired glucose tolerance; NGT = normal glucose tolerance; PGT = pathological glucose tolerance; and PPPD = pylorus-preserving partial pancreatoduodenectomy

In total, 32% of patients older than 60 years displayed improved glucose homeostasis after pancreatic resection, while only 5% of patients younger than 60 years improved (Table 1). This finding can be explained by a strong association between age and the underlying disease. Of the 24 patients with chronic pancreatitis, 22 (92%) were younger than 60 years, while 37 of the 45 (82%) patients with malignant tumors and 5 of the 15 (33%) patients with benign tumors were older than 60 years (p <.001).

Postoperative changes in glucose-homeostasis category were mainly observed if the diagnosis of glucose dysregulation was made less than 6 months before surgery. Specifically, 96% of patients with a long-standing (>6 months) diagnosis of glucose dysregulation remained stable three months after surgery. In contrast, 32% of patients with short-standing impairment of blood glucose homeostasis were stable, while 23% deteriorated and 45% improved (p<.001; Table 1). Notably, we did not observe a clear association between the duration of glucose dysregulation and the underlying disease. Specifically, 9/15 (60%) patients with CP were diagnosed <6 months before surgery, while this was the case for 16/34 (47%) patients with malignant tumors and 6/7 (86%) patients with benign tumors (p = .16).

Furthermore, interdependence was determined between the pre- and postoperative glucose homeostasis category and glucose dynamics. The majority (91%) of initially diabetic patients (category 4: elevated fasting glucose and/or anti-diabetic medication) remained stable after surgery. In contrast, patients with preoperative IGT or PGT exhibited a highly dynamic status after surgery, with only 24% and 30% remaining stable, respectively (p<.001; Table 1). Indeed, it is remarkable that the majority (25/32; 78%) of the diabetic patients, defined as those with elevated fasting glucose and/or taking anti-diabetic medication, had a long-standing diagnosis of diabetes, specifically, >6 months prior to surgery. In contrast, in all of the patients with IGT or PGT, glucose dysregulation was uncovered by the preoperative oGTT analysis (p<.001).

Importantly, no relevant interrelations were determined between post-pancreatectomy dynamics in glucose metabolism and surgical procedure, gender, pre-operative weight loss >5 kg or pancreatic duct dilatation. However, none of the patients who underwent pancreatic left resection improved after surgery.

### Continuous variables

#### HbA1c levels and onset of blood glucose dysregulation

**Fig 1:**
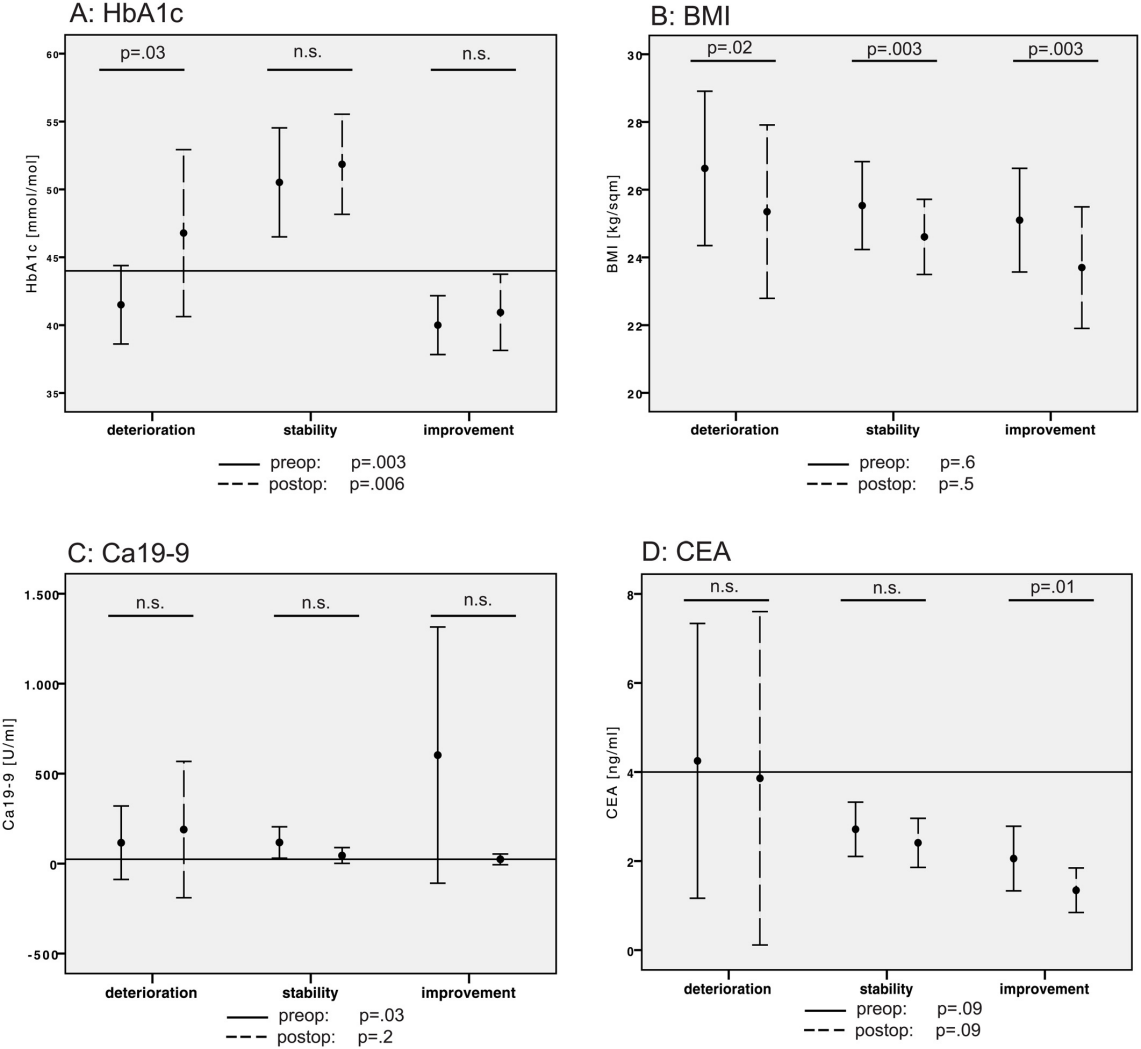
Bar graphs of the pre- and postoperative values for (A) HbA1c, (B) BMI, (C) Ca19-9 and (D) CEA with respect to deteriorated, stable and improved blood glucose homeostasis. Points: means; error bars: 95% CI; black error bars: preoperative values; broken error bars: postoperative values for each group; reference lines: cut-off/normal values; F- and p-values beneath each diagram: one-sided ANOVA for comparison between the three groups at each time point; and p-values within the diagram: two-sided T-test for paired samples for pre- and post-operative comparisons within the designated group.

Analysis of the categorical data suggests that, in addition to the histological diagnosis, the duration of blood glucose dysregulation may be an important determinant of the postoperative dynamics of glucose metabolism. This finding was confirmed by the time course of HbA1c levels. Specifically, the stable group displayed elevated HbA1c levels compared with the deterioration and improvement groups before surgery (p = .003), and with the improvement group after surgery (p = .006). Three months after surgery, the HbA1c levels deteriorated in the deterioration group (p = .03), while no changes were observed in the other groups (Fig 1A). These results point to a temporary blood glucose dysregulation in the improvement group.

#### Body weight dynamics

All three groups exhibited a slight decrease in their BMIs three months after the operation, whereas the groups did not differ in their BMIs pre- or post-operatively (Fig 1B). Accordingly, BMI dynamics are unlikely to determine the postsurgical blood-glucose dynamics.

#### Tumor marker dynamics

The analysis of tumor markers revealed pathological Ca19-9 levels in the improvement group before surgery (p = .03), with a clear tendency toward postoperative normalization. In contrast, the deterioration group displayed similar Ca19-9 serum levels before and after surgery (Fig 1C). CEA levels were in the physiological range before and after surgery in all three groups, but were reduced significantly after surgery in the stability group (p = .01; Fig 1D).

#### Dynamics of the pancreatic, bile duct and liver cell parameters

**Fig 2:**
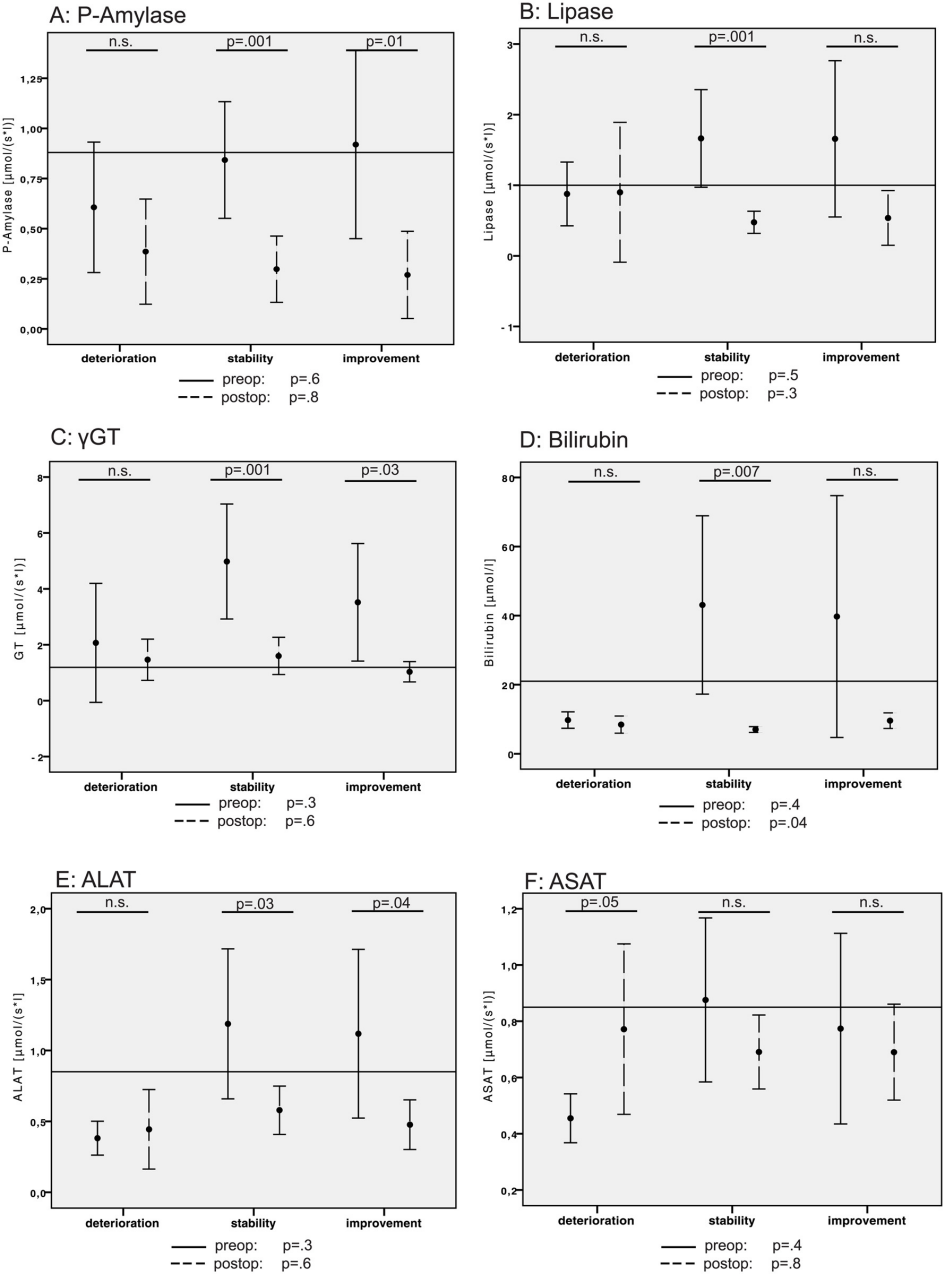
Bar graphs of the pre- and postoperative values for (A) amylase, (B) lipase, (C) γGT, (D) bilirubin, (E) ALAT and (F) ASAT with respect to deteriorated, stable and improved blood glucose homeostasis. Points: means; error bars: 95% CI; black error bars: preoperative values; broken error bars: postoperative values for each group; reference lines: cut-off/normal values; F- and p-values beneath each diagram: one-sided ANOVA for comparison between the three groups at each time point; and p-values within the diagram: two-sided T-test for paired samples for pre- and post-operative comparisons within the designated group.

The serum concentrations of the pancreatic enzymes P-amylase and lipase (Fig 2A and 2B) and bile duct obstruction parameters γGT and bilirubin (Fig 2C and 2D) displayed similar dynamics. In the deterioration group, normal or near-normal values were observed before and after surgery, whereas in the stability and improvement groups, postoperative normalization of the initially pathological values was observed. Notably, in terms of liver cell damage parameters (Fig 2E and 2F), liver-specific ALAT normalized in the stable and improvement groups (p = .03 and p = .04), while the globally distributed transaminase, ASAT, worsened in the deterioration group (p = .05) but did not change significantly in the other two groups.

#### Negative findings

No relevant association was found between blood glucose homeostasis dynamics and inflammatory markers (CRP/leukocytes), serum albumin, total protein or lipid profile (triglycerides/HDL/LDL/total cholesterol).

### Insulin resistance and insulin secretion

**Fig 3:**
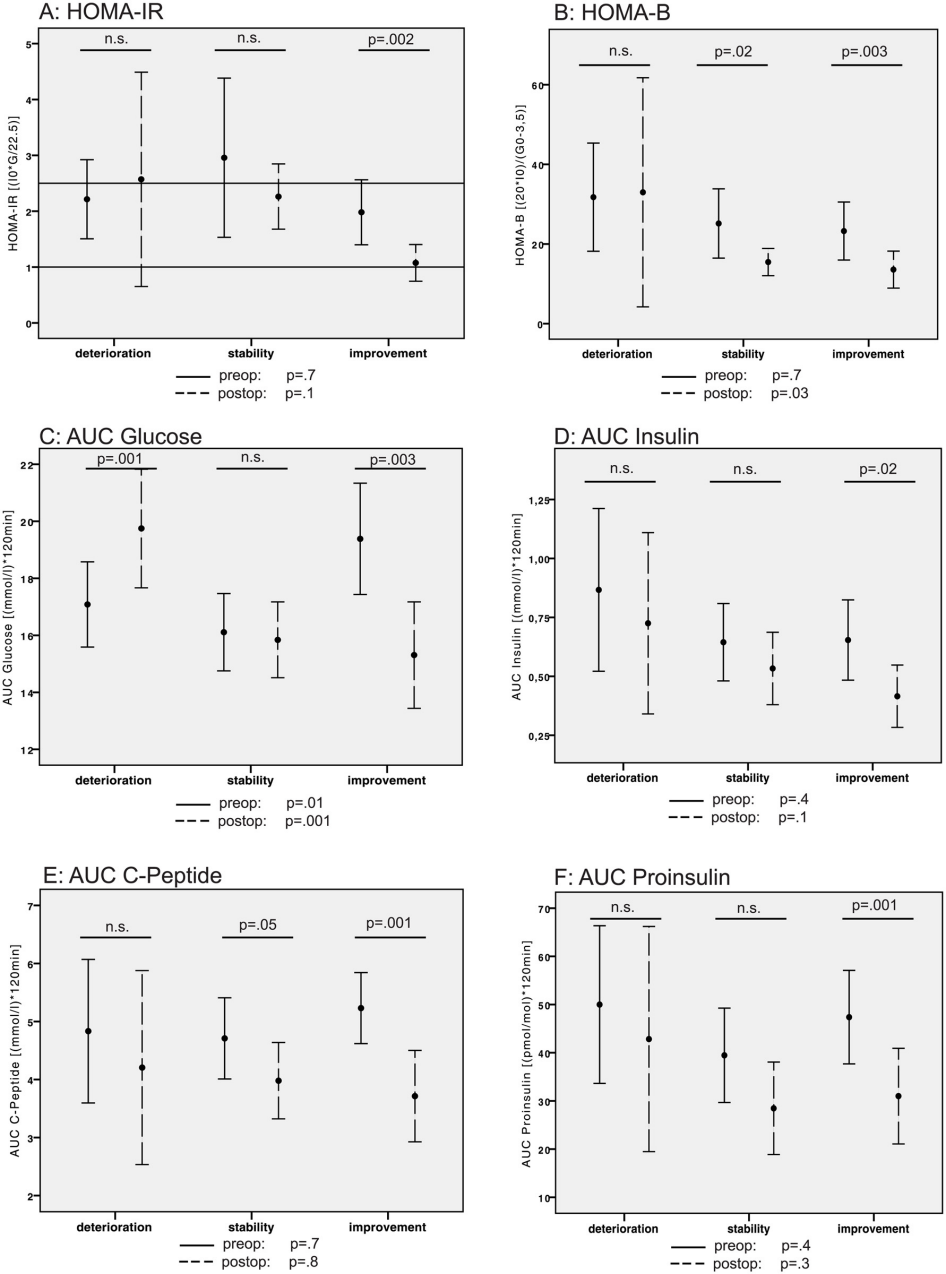
Bar graphs of the pre- and postoperative values for (A) HOMA-IR, (B) HOMA-B, and AUCs (C) for glucose, (D) insulin, (E) C-peptide and (F) proinsulin with respect to deteriorated, stable and improved blood glucose homeostasis. (Points: means; error bars: 95% CI; black error bars: preoperative values; broken error bars: postoperative values for each group; reference lines: cut-off/normal values; F- and p-values beneath each diagram: one-sided ANOVA for comparison between the three groups at each time point; and p-values within the diagram: two-sided T-test for paired samples for pre- and postoperative comparisons within the designated group).

Assessment of the insulin resistance index based on the fasting blood glucose and insulin serum levels (HOMA-IR) did not reveal differences among the groups at each time point. However, a marked decrease in the HOMA-IR values was detected after three months in the improvement group (p = .002). The HOMA-IR values in the deterioration group displayed instead a tendency toward increment, while a slight, albeit not significant, decrease in HOMA-IR was noticed in the stability group three months after the operation (Fig 3A). Insulin secretion in the fasting state, which is reflected in the HOMA-B index, decreased in the improvement (p = .003) and stability (p = .02) groups, but remained constant over time in the deterioration group (Fig 3B).

In total, 52 participants with normal fasting glucose and without anti-diabetic treatment underwent pre- and/or post-operative oGTT. The AUCs were calculated for glucose, insulin, C-peptide and proinsulin. As expected, the AUCs for glucose were different at each time point (pre- and post-operative) among the groups. Preoperatively, elevated AUCs for blood glucose values were observed in the improvement group, compared to the others (p = .01). Postoperatively, elevated AUCs for blood glucose were only observed in the deterioration group (p = .001). At both time points, a significant increase in the AUC for glucose was observed in the deterioration group (p = .001), while a decrease was observed in the improvement group (p = .003, Fig 3C).

The pre- and post-operative AUC measurements for insulin, C-peptide and proinsulin did not differ among the groups at each time point. However, tendencies for decreased postoperative insulin, C-peptide and proinsulin secretion were observed in all three groups, and most markedly, in the improvement group. Specifically, in the improvement group, the AUCs for insulin (p = .02, Fig 3D), C-Peptide (p = .001, Fig 3E) and proinsulin (p = .001; Fig 3F) decreased three months after the operation, pointing to a decreased requirement for insulin secretion secondary to improved blood glucose control.

## Discussion

The deterioration of blood glucose homeostasis might be expected after surgical β-cell mass reduction. Based on different observations, however, other hypotheses have been proposed concerning the interdependence between pancreatic pathologies and diabetes. First, chronic pancreatitis can lead to inflammatory mediated damage of β–cells and, thus, to pancreatic (also defined as type 3c) diabetes [21]. Second, diabetes is a predisposing factor for pancreatic malignancy. This notion is mainly based on the high co-incidence of diabetes or disturbed glucose homeostasis with pancreatic cancer [7]. Third, a specific but undetermined type of pancreatic malignancy may induce diabetes via unknown factors produced by the tumor because cancer resection can lead to the resolution of diabetes [22–24]. To evaluate the influence of the numerous potential co-factors associated with relevant dynamics of blood glucose homeostasis in the course of pancreatic resection, the present observational study included a broad spectrum of (peri-) pancreatic pathologies and surgical approaches.

In our cohort, two thirds of the participants displayed disturbed glucose control before the operation. A long-standing diagnosis of diabetes regularly resulted in unaltered glucose control after pancreatic resection, while subjects with blood glucose disturbances diagnosed concomitantly with the pancreatic pathology displayed dynamic glucose control. This observation alone suggests that categorical differences exist between pre-existing “diabetes *per se*” and a surgically reversible glucose metabolism disorder secondary to pancreatic pathology.

The analysis of variables associated with different outcomes in blood glucose regulation shed light on the nature of this surgically reversible glucose metabolism disorder. Comparable with previous studies [1,9,22,25–27], more than 80% of participants showed stable or even improved blood glucose homeostasis, despite surgical reduction in pancreatic tissue and thus, conceivably, of β–cell mass. In particular, improving patients were characterized by the following: (1) presence of a (preferentially malignant) pancreatic tumor, (2) diagnosis of impaired glucose homeostasis in the oGTT, but rarely elevated fasting glucose, concomitantly with tumor diagnosis, (3) postoperative normalization of insulin resistance and (4) postoperative decreased insulin secretion in the fasting and glucose-stimulated state. For patients with chronic pancreatitis or who underwent pancreatic left resections, improvements were not detected. However, improvements were detected in patients who underwent head resections for tumor removal.

Remarkably, improving patients were characterized by initially elevated and postoperatively normalized levels of serum markers for tumor presence, acute pancreatitis, common bile duct obstruction and liver cell damage. Using the same serum markers (AP, γGT, ALAT, ASAT) in the context of chronic liver diseases, the evidence for increased insulin resistance and type 2 diabetes induced by bile duct and liver cell damage is compelling [11–17]. Thus, our observations strongly suggest that a surgically reversible form of glucose metabolism disorder exists, which is most likely secondary to obstruction of the bile duct by a tumor in the head of the pancreas, with subsequent impairment of liver function.

Relying on a minimalistic but practical assessment of glucose metabolism is a limitation of our study. More sophisticated tools, including hyperglycemic or euglycemic clamps and intravenous glucose tolerance tests, can be used to obtain data of high quality for the quantification of islet function, insulin action and potential influence of anatomic changes in the upper gastrointestinal tract. However, excellent correlations between clamp tests and the HOMA model for the quantification of insulin resistance have been consistently reported in cohort studies [28]. Furthermore, the scope of our study was to screen severely ill individuals for clinically relevant changes in glucose metabolism. As such, additional hospital admissions for clamp testing were not ethically justifiable, while oral glucose tolerance tests were both convenient for patients and sufficient for detection of clinically relevant changes.

Another limitation of this study is the lack of quantification of islet cell reduction by the surgical intervention. However, the feasibility of estimating the extent of islet mass reduction upon pancreatic resection is questionable for various reasons. The extent of surgical islet cell reduction is not likely to be a linear function of the extent of parenchyma reduction because islets are not evenly distributed throughout the gland and islet density in the tail is higher than in the head [29]. As such, pancreatic parenchyma reduction must be individually adapted to the underlying disease. The influence of (frequently existing) pancreatic inflammation and its distribution within the gland on islet mass and function is also not quantifiable. Although partial pancreatectomy can induce the regeneration of islet cells in murine models [30], solid evidence for comparable regeneration in humans is lacking [31]. To overcome these issues, complete histological work-up with quantitative assessments of the histo-morphology of the resected specimen might be a potential, but unrealistically demanding, tool for estimating the mass of removed islet cells. However, even so, this approach would not yield any information about the function and mass of islets located in the residual gland.

Regardless, our data robustly challenge the notion of a relevant influence of islet cell mass or function on advantageous dynamics of glucose control after partial pancreatic resection. On the contrary, decreased basal insulin secretion was detected in patients with stable and improved glucose control, while improvement was accompanied by a pronounced decrease in β-cell secretion during oGTT. Thus, the decrease in insulin resistance is key for stable and improved glucose control, while the reserve of islet cell mass after resection is sufficient for adequate insulin secretion and glucose control. Conceivably, increased preoperative insulin resistance leads to an adaptive increase of insulin secretion, which reverses after the removal of cholestasis and normalization of insulin resistance.

Deterioration was accompanied by a non-significant decrease in insulin secretion and a non-significant increase in insulin resistance. No relevant associations between the analyzed co-variables and deterioration were found. Therefore, our results cannot conclusively explain deteriorating glucose control after pancreatectomy, and this is most likely due to the small sample size of this subgroup.

Unexpectedly and in contradiction to the characteristics of bariatric surgery, decreased incretin responses in association with stable and improved glucose control were described after PPPD, but not after distal pancreatectomy [9]. This observation agrees completely with our results of decreased insulin secretion in the stable and improvement group. A decrease of incretin and insulin secretion after PPPD is a plausible mechanism of adaption to the normalized liver-mediated insulin resistance after bile flow restoration.

A weak point of our study is the small number of duodenum-preserving procedures and pancreatic left resection. Thus, the results are insufficient to conclusively verify or exclude an influence of altered anatomy of the gastro-duodeno-jejunal passage on glucose control. However, no pancreatic left resections, and only a minority of duodenum-preserving procedures (8%), resulted in improved glucose control, while approximately one third of PPPDs led to improvement. Wu and colleagues previously observed diabetes resolution after PPPD for both the removal of PDACs and non-PDACs. Therefore, the authors attributed improved glucose control to anatomical changes in the upper gastrointestinal tract and not to a PDAC-intrinsic diabetogenic factor [1]. Unfortunately, liver function parameters were not measured. Nevertheless, our data corroborate the independence of improved glucose control from a specific tumor entity and therefore, suggest that rather than tumor characteristics, the restoration of bile flow and normalization of liver function most likely play key roles in improved glucose control. Anatomical alterations of the gastro-duodeno-jejunal passage can inevitably restore bile flow and improve liver function in cases of decreased insulin resistance.

Taken together, the results of this observational study suggest the existence of a reversible glucose metabolism disorder, specifically, diabetes, which is secondary to tumor obstruction of the common bile duct and must be categorically discerned from other forms of diabetes. It is characterized by pathological oral glucose tolerance (i.e., not frank diabetes), often coincides with tumor diagnosis and is independent of the tumor entity. Instead, the increased insulin resistance is most likely secondary to liver cell damage and reversible upon tumor resection. These data enable the pancreatic surgeon to estimate the postoperative metabolic consequences in the course of partial pancreatic resection and to improve his patient’s information accordingly. Future studies dealing with “diabetes” as a potential risk factor for malignancies must take into account that the tumor can cause hyperglycemia due to liver cell damage. Finally, “new-onset diabetes” in elderly patients (age >50 years) was previously proposed as a first filter for pancreatic cancer screening. Unfortunately, a specific biomarker for selecting screening candidates, which discerns pancreatic cancer-associated hyperglycemia from other forms of diabetes, was not observed [2]. Based on our results, temporary surveillance of bile duct, pancreas and liver cell parameters during the first months after the diagnosis of impaired glucose metabolism in the elderly might serve as an effective and practical second filter for pancreatic cancer screening. Of course, the implementation of such an approach requires further validation in prospective, population-based trials. Finally, future in-depth “omic” studies on blood and islet specimen from partially pancreatectomized patients with reversible diabetes secondary to cholestasis may allow the identification of circulating biomarkers and adaptive traits of beta cells ensued by an acute onset of insulin resistance.
